# Guided current-induced skyrmion motion in 1D potential well

**DOI:** 10.1038/srep10620

**Published:** 2015-05-29

**Authors:** I. Purnama, W. L. Gan, D. W. Wong, W. S. Lew

**Affiliations:** 1 School of Physical & Mathematical Sciences, Nanyang Technological University, 21 Nanyang Link, Singapore 637371

## Abstract

Magnetic skyrmions are particle-like magnetization configurations which can be found in materials with broken inversion symmetry. Their topological nature allows them to circumvent around random pinning sites or impurities as they move within the magnetic layer, which makes them interesting as information carriers in memory devices. However, when the skyrmion is driven by a current, a Magnus force is generated which leads to the skyrmion moving away from the direction of the conduction electron flow. The deflection poses a serious problem to the realization of skyrmion-based devices, as it leads to skyrmion annihilation at the film edges. Here, we show that it is possible to guide the movement of the skyrmion and prevent it from annihilating by surrounding and compressing the skyrmion with strong local potential barriers. The compressed skyrmion receives higher contribution from the spin transfer torque, which results in the significant increase of the skyrmion speed.

Skyrmions, which were initially used to describe baryons in particle physics^1^, have recently been observed in extended lattices of bulk non-centrosymmetric magnetic materials such as MnSi, (FeCo)Si or FeGe^2^,^3^,^4^. The skyrmions have also been observed via spin-polarized scanning tunneling microscopy (STM) at zero applied fields in Fe monolayer that is grown on Ir (111)^5^. The presence of skyrmions in such ultrathin film is attributed to the Dzyaloshinskii–Moriya (DM) interaction^6^,^7^, which arises from a three-site indirect exchange mechanism between two spins and a neighbour atom with a large spin-orbit coupling. The DM energy term (**H**_DM_) is denoted by
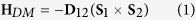
where **D**_12_ is the DM vector while **S**_1_ and **S**_2_ represent the two spins at the interface. The DM vector is perpendicular to the unit vector joining **S**_1_ and **S**_2_ and lies on the interface plane. The spins in the magnetic film are then tilted from their easy axis to form the skyrmion as the DM interaction tries to align the spins perpendicular to each other. Due to its topological configuration, the skyrmions have been shown to be far less hindered by defects when driven by current^8^,^9^,^10^,^11^. However, the skyrmion is also driven away from the conduction electron flow direction due to the presence of the Magnus force^12^,^13^, which leads to skyrmion annihilation at the film edges^14^.

Here, we show that it is possible to counter the Magnus force by surrounding the skyrmion with potential barriers. We also show that the speed of the current-driven motion of the skyrmion can be increased significantly by narrowing the space between the potential barriers and compressing the skyrmion at the same time. The increase in the speed can be attributed to the increased effectiveness of the spin-transfer torque (STT) that the compressed skyrmion receives from the conduction electron.

## Skyrmion motion in a wide plane

**Figure 1:**
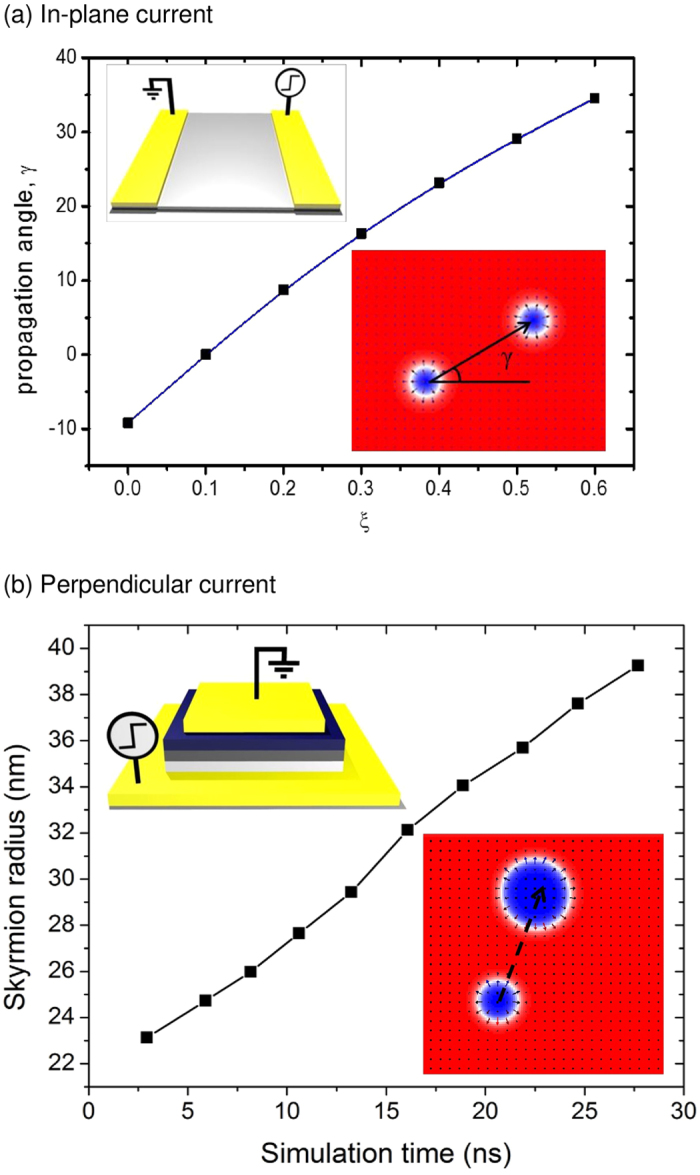
Skyrmion motion in a wide plane (**a**) The change in the propagation angle of the skyrmion as a function of the non-adiabatic constant. Top inset is a schematic of a setup for skyrmion driving with in-plane current. Bottom inset is a snapshot of a skyrmion driving simulation with in-plane current (**b**) The change in the size of the skyrmion as a function of time. Top inset is a schematic of a setup for skyrmion driving with perpendicular current. Bottom inset is a snapshot of a skyrmion driving simulation with perpendicular current

Inset of Fig. 1(a) shows the movement of a skyrmion in a wide plane under the application of an in-plane current for various Gilbert damping (*α*) and non-adiabatic STT constants (*ξ*). In the in-plane driving case, the magnetization dynamics is expressed by the modified Landau-Lifshitz-Gilbert (LLG) equation (see Eq. 6 in Methods section), and the skyrmion is driven mostly by the field-like torque from the STT^15^,^16^. The simulation results show that under the application of in-plane current, the skyrmion moves at an angle with respect to the conduction electron flow when *α* ≠ *ξ*^17^. Figure 1(a) shows the change in the propagation angle as a function of the non-adiabatic constant. The movements of the skyrmion away from the intended direction can be attributed to the presence of the Magnus force, which arises due to the coupling between the conduction electron and the local magnetization^12^,^13^. The equation for the drift velocity of the skyrmion can then be obtained by mapping the LLG equation onto the translational mode in the continuum limit, while assuming the rigidity of the spin textures during the skyrmion motion^12^. The skyrmion drift velocity then reads:
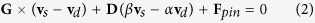
where **v**_d_ is the drift velocity of the skyrmion while **v**_s_ is the velocity of the conduction electron. The first term in the left hand side of Eq. 2 is the Magnus force with **G** as the gyromagnetic coupling vector; the second term is the dissipative force with **D** as the dissipative force tensor; and lastly the third term is the phenomenological pinning force (**F**_pin_) due to impurities. *β* is the non-adiabatic constant of the STT as expressed by Thiaville (See Methods)^16^. In the absence of impurities (**F**_pin_ = 0), the drift velocity of the skyrmion is expressed as:
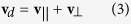

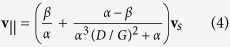

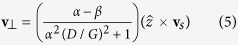
where **v**_**||**_ and **v**_⊥_ are the components of **v** parallel and transverse to **v**_s_, respectively.

We found that the skyrmion is also deflected from the intended direction when it is driven under perpendicular current injection. Inset of Fig. 1(b) shows the schematic and the snapshot of the micromagnetic simulations of the skyrmion driving with perpendicular current. The perpendicular current injection can be achieved by using the setup of magnetic tunnel junction (MTJ) or spin hall devices^18^,^19^, with the magnetization of the reference layer fixed along the *x* or *y* axis. Under the injection of perpendicular current, the skyrmion is now driven mostly by the torque from the angular momentum transfer as compared to the field-like torque of the STT^15^. As a result, the skyrmion is able to move with a considerably higher speed due the angular momentum transfer torque being much stronger than the field-like torque. The LLG equation which describes the magnetization dynamics under the application of perpendicular current is expressed in Eq. 11 (see Methods)^15^,^20^. The skyrmion is also shown to increase in size as it moves along the plane. Figure 1(b) shows the change in the skyrmion size as a function of simulation time as it moves along the ultrathin magnetic layer. See Supplementary Material for details of the skyrmion driving under perpendicular current injection.

## Skyrmion motion under the presence of local potential barriers

**Figure 2:**
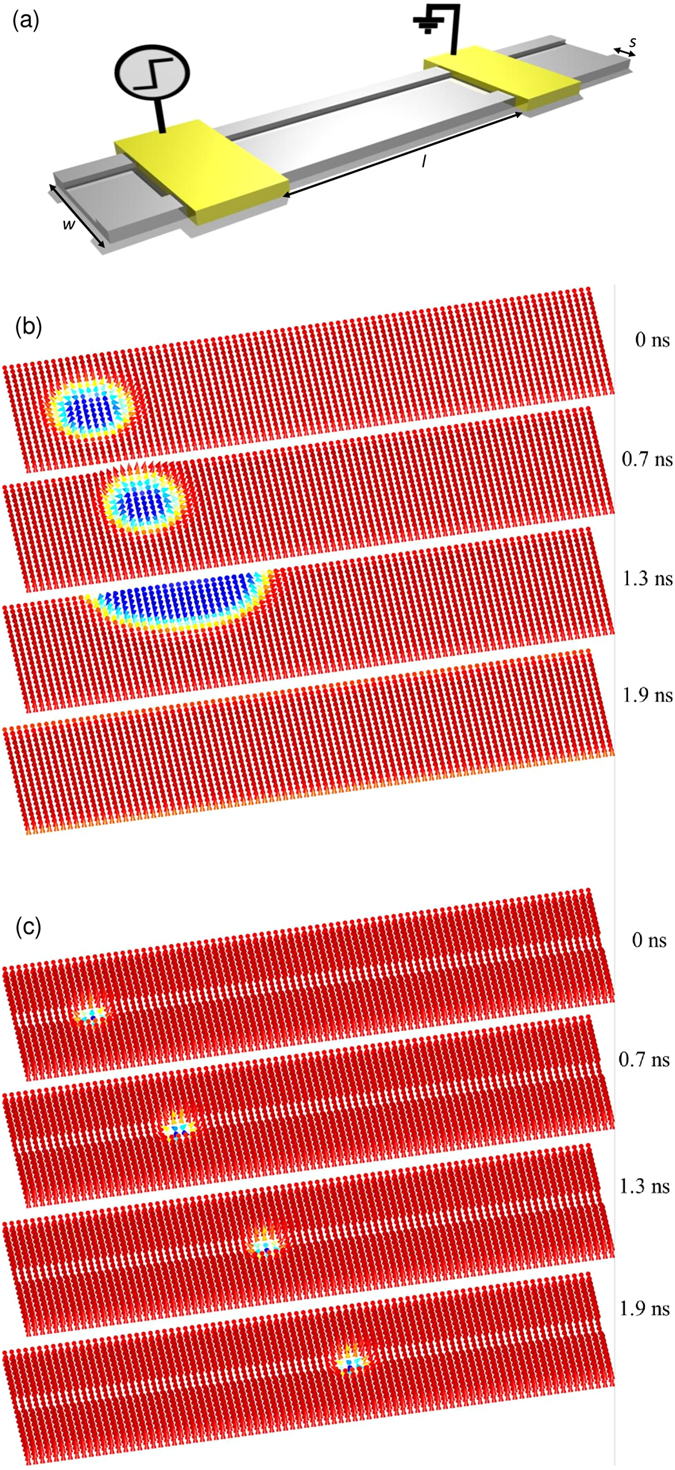
Proposed kerbed track (**a**) Schematic of the proposed kerbed skyrmion track (**b**) Snapshot of the skyrmion driving simulation on a track without kerbs which results in skyrmion annihilation. (**c**) Snapshot of the skyrmion driving simulation on a kerbed track which results in fast skyrmion motion.

As discussed above, when the skyrmion is driven on a track it deviates from the intended propagation direction after a short travelling distance because of the Magnus force. The deflection towards the edge of the track eventually leads to skyrmion annihilation. The deviation of the skyrmion motion from the conduction electron flow also prevents reversible two-way operation, as the skyrmion tend to move to the opposite edge of the track when the applied current is reversed. Figure 2(a) shows the schematic of our proposed kerbed track that shall impose a stronger edge potential barrier on the skyrmion^21^. The kerbed track is formed by creating a rectangular groove on the surface of the perpendicular magnetization anisotropy (PMA) nanowire, leaving the two edges of the nanowire with limited height and width. The kerb structures function to confine the skyrmion within the PMA grove. Figure 2(b,c) show snapshots of simulation with a skyrmion within the unkerbed device and kerbed device, respectively. If we consider each of the kerbs to have a width of *s*, the diameter (*d*) of the skyrmion is then forcibly shrunk into *d* = *w −* 2*s*, where *w* is the width of the ferromagnetic layer. At *t* = 0, an in-plane current is applied to a skyrmion in the relaxed state on both the unkerbed and kerbed tracks. At *t* = 0.7 ns, the skyrmion on the unkerbed track is shown to be pushed towards the edge of the track while the skyrmion on the kerbed track is shown to propagate further. At *t* = 1.3 ns, the skyrmion on the unkerbed track is approaching the edge of the track and it starts to get annihilated. Eventually, the skyrmion on the unkerbed track is annihilated completely at *t* = 1.9 ns. In contrast, the skyrmion on the kerbed track is stable and the propagation is well guided without any sign of deflection. A movie which shows the motions of the skyrmion on both the unkerbed and kerbed tracks is included as Supplementary Information, Video 1.

**Figure 3:**
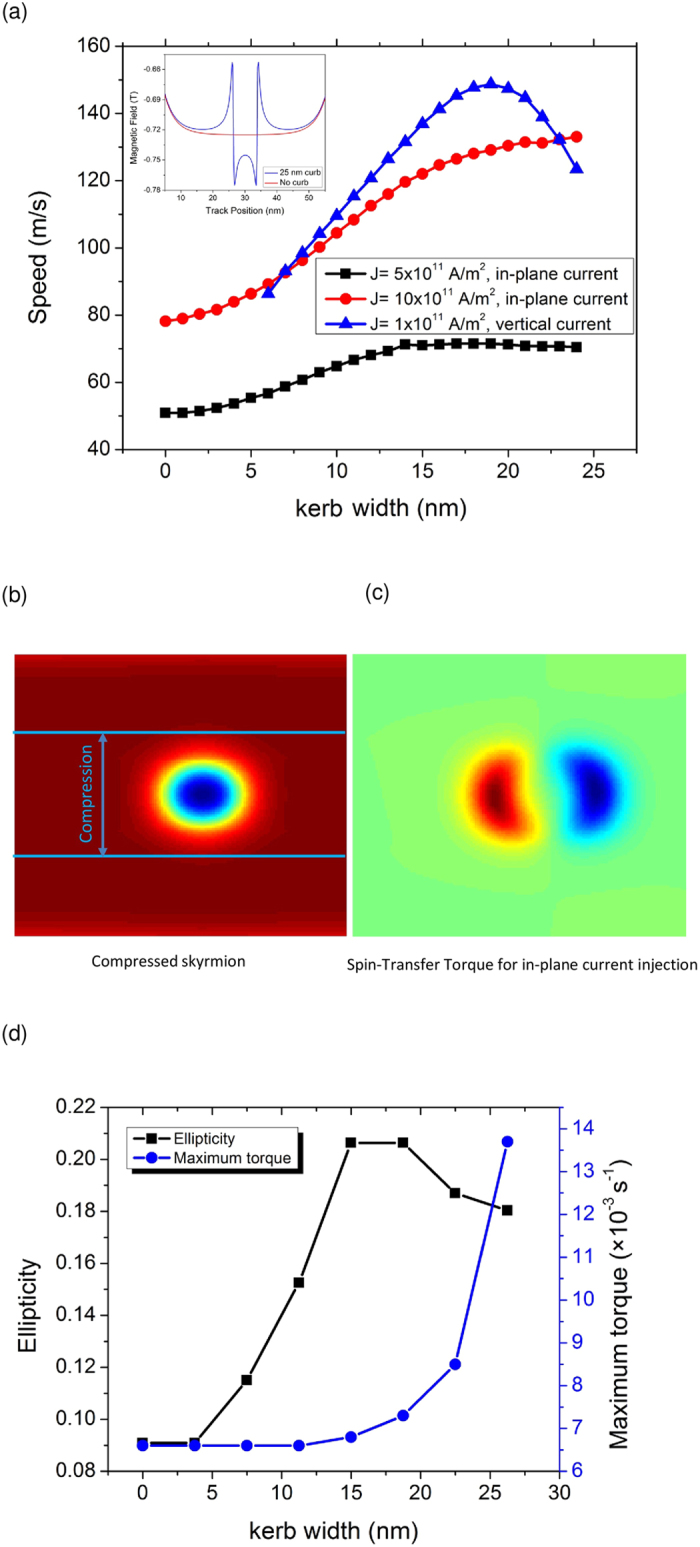
Fast skyrmion motion on the kerbed track (**a**) The skyrmion speed as a function of the kerb width for various applied current density. Inset is a cross-sectional plot of the kerbed track to show the stray field that is generated by the kerbs. (**b**) Snapshot of the compressed skyrmion on the kerbed track. (**c**) The associated spin-transfer torque that is acting on the skyrmion when an in-plane current is applied. (**d**) The elipticity of the skyrmion and the maximum torque that the skyrmion receives as functions of kerb width.

Figure 3(a) shows the speed of the skyrmion in the proposed kerbed device. The results show that the skyrmion speed is increased due to the inclusion of the kerbs, for both in-plane driving setup and also perpendicular driving setup. For instance, when the skyrmion is driven in the in-plane driving setup with a current density of *J* = 10 × 10^11^ A/m^2^ and kerbs of *s* = 15 nm wide, the speed of the skyrmion is found to be increased by almost 50%, from 75 m/s to 110 m/s. The speed increment is found to be more prevalent in the case of the perpendicular driving setup. For instance, the speed can be increased to as much as 130 m/s by using the same kerbs at just a tenth of the current density that is used in the in-plane driving setup (*J* = 1 × 10^11^ A/m^2^). Inset of Fig. 3(a) shows the stray magnetic field that the kerbs exert to the skyrmion. The stray field from the kerbs is directed in the same direction as the skyrmion magnetization, hence the stray field acts to increase the stability of the skyrmion against thermal fluctuation^22^.

The increase in skyrmion driving speeds can be attributed to the increased STT that the skyrmion receives when the width of the kerbed track is less than the original diameter of the skyrmion. Figure 3(b) shows a snapshot of a skyrmion that is compressed by the kerbs at the two edges as the skyrmion can only exist within the area that is not covered by the kerb. As a result, the skyrmions become elliptical and elongated along the nanotrack axis. Figure 3(c) shows the STT that acts on a skyrmion in the case of in-plane driving. The calculated torque is shown to be most prominent at the front and the back sides of the skyrmion, where the front torque is directed at *–z* while the back torque is directed at +*z*. The two torques are equal in values with a maximum value of *τ*_drive_ = 6.5 × 10^−3^ s^−1^ when the skyrmion is driven on an unkerbed track. Figure 3(d) shows the ellipticity of the skyrmion and the maximum torque (*τ*_drive_) as functions of the kerb width (*s*). The maximum torque that acts on the skyrmion is found to increase rapidly with increasing kerb width, which results in the increased skyrmion speed. The increased torque can be attributed to the fact that the compressed skyrmion possesses a higher magnetization divergence, which directly affects the STT, as shown in Eq. 7.

**Figure 4:**
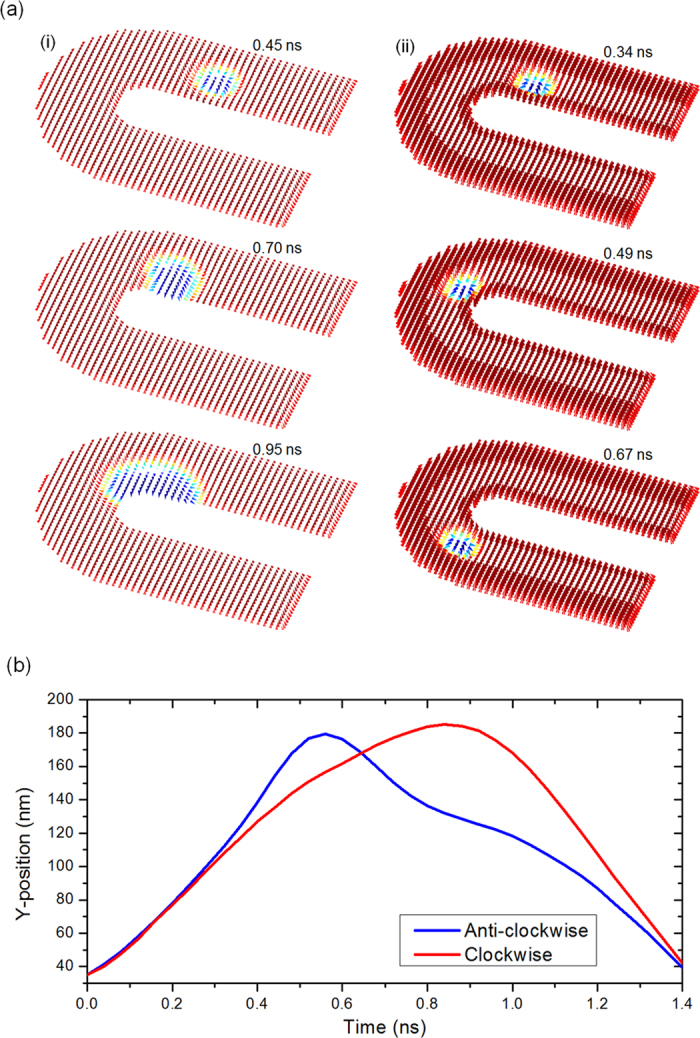
Guided skyrmion motion on a curved track **a**) Skyrmion motion on a curved track (i) without kerbs and (ii) with kerbs. (**b**) Skyrmion position along the *y* axis as a function of time for both clockwise driving and anti-clockwise driving.

It is possible to extend the skyrmion guiding technique to drive the skyrmion on a curved track. Figure 4(a) shows the snapshots of the simulations when the skyrmion is driven on a curved track which has a 180^0^ turning angle. The results show that without the kerb (i), the skyrmion is annihilated within the curvature even though the current is distributed to follow the shape of the track. However, with the inclusion of the kerbs to the track (ii), the annihilation is prevented and the skyrmion is guided to make the 180^0^ turn. Figure 4(b) shows the position of the skyrmion within the kerbed curved track as a function of time in the case of clockwise and anti-clockwise driving. In contrast to the straight track where the skyrmion maintains a constant velocity throughout its motion, the skyrmion on the curved track is shown to be slowed down with different deceleration depending on the direction of the applied current. The slowing down of the skyrmion can be attributed to the uneven current distribution that the skyrmion receives within the curved track, while the different in the behavior between the clockwise and anti-clockwise driving can be attributed to the Magnus force. As discussed before, during the application of current, the skyrmion is pushed to one side of the track due to the Magnus force. Therefore, with the application of clockwise and anti-clockwise currents, the skyrmion is pushed to the outer and inner arc, respectively. Since the turning radius for the inner arc is much smaller, it is more difficult for the skyrmion to complete the turn when it is driven in the anti-clockwise direction. A movie which shows the motions of the skyrmion within the curved track for both clockwise and anti-clockwise driving is included as Supplementary Information, Videos 2 and 3.

**Figure 5:**
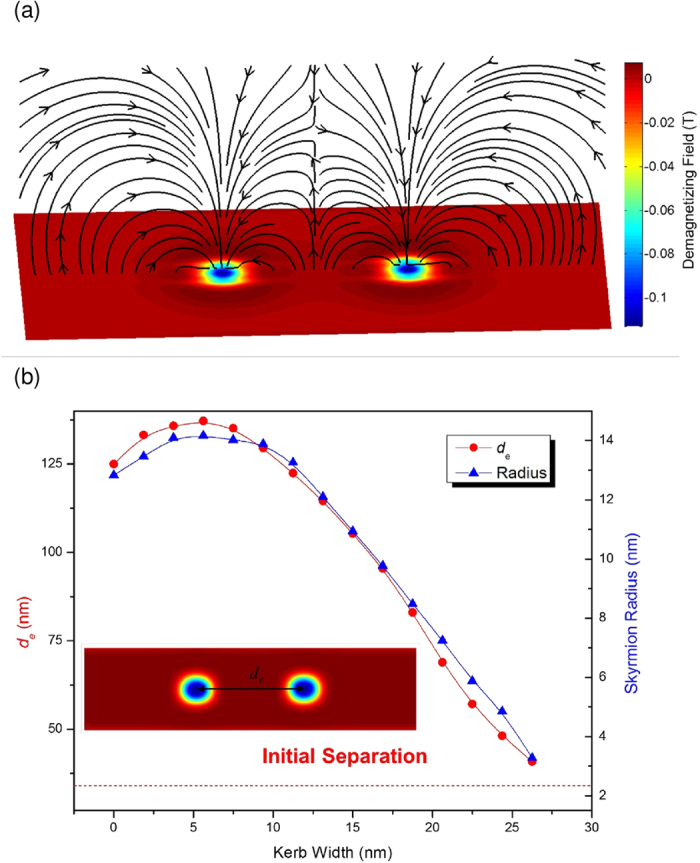
Skyrmion-skyrmion interaction on the kerbed track (**a**) The stray magnetic fields that that are created around two interacting skyrmions. (**b**) Plot showing the relation between the equilibrium distance (*d*_*e*_), the skyrmion radius, and the kerb width.

The reduced size of a skyrmion can also confer a significant increase in data storage density to a potential skyrmion-based memory device. As skyrmions in a magnetic thin film are of the same charge, two skyrmions experience repulsion from each other, and the strength of the repulsion is related to the size of the skyrmions, as shown by Fig. 5(a). The minimum inter-skyrmion distance whereby two skyrmions can remain undisturbed from each other therefore shall also depend on the skyrmion size. Our simulations show that the inter-skyrmion distance (*d*_e_) and the radius of the skyrmion (*r*_s_) varies almost linearly, as shown in Fig. 5(b). This means that the kerbed track can also be utilized to pack the skyrmions closer to each other. At kerb widths of around 5 nm, we observed that the skyrmion was unexpectedly enlarged. This behavior can be attributed to the presence of the stray magnetic field from the kerb as explained previously. However, the size increment from the stray magnetic field is immediately overcome by the skyrmion compression from the kerbs as the distance between the kerbs is decreased.

In conclusion, our simulation results have shown that it is possible to guide the skyrmion motion and prevents the skyrmion to be annihilated at the same time by creating kerbs around the skyrmion to counter the Magnus force. We show that the technique can be extended to drive the skyrmion on a curved track, with different speed depending on the direction of the applied current. We also show that the speed of a current-driven skyrmion is increased significantly when the skyrmion is compressed by the kerbs. The increase in the current-driven speed can be attributed to the higher torque that the compressed skyrmion receives from the conduction electron.

## Methods

### Micromagnetic simulations

We performed micromagnetic simulations using the Mumax program^17^,^20^,^23^. The magnetization dynamics in the case of in-plane current injection is expressed by^24^,^25^,^26^:
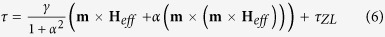

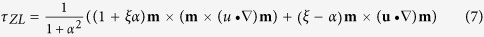

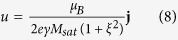
where **m** is the normalized unit vector of the local magnetization, γ is the electron gyromagnetic ratio, **H**_eff_ is the effective field, *α* is the Gilbert damping constant, *e* is the elementary charge, *M*_*sat*_ is the saturation magnetization, **j** is the current density vector, while *ξ* is the degree of the non-adiabacity.

Eq. (6) is expressed in another form by Thiaville^16^,^23^

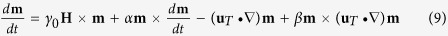

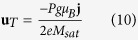


Additional simulations were performed by using the OOMMF program^27^ and similar skyrmion dynamics were observed^17^,^23^.

The magnetization dynamics in the case of perpendicular current injection is expressed by:
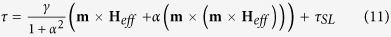

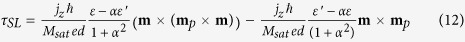

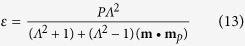
where *j*_z_ is the current density along the *z* axis, d is the skyrmion layer thickness, **m**_**p**_ is the fixed layer magnetization, *P* the spin polarization, the Slonczewski *Λ* parameter characterizes the spacer layer, and *ε*’ is the secondary spin torque parameter.

The material parameters that were chosen for the simulations correspond to Co/Pt multilayers^28^,^29^. The values are as follow: exchange stiffness = 15 × 10^−12^ J/m, saturation magnetization (M_sat_) = 580 × 10^3^ A/m, Gilbert damping (*α*) = 0.1, DMI strength (*D*) = 3 × 10^−3^ J/m^3^, non-adiabacity of STT (*ξ*) = 0.35, magnetocrystalline anisotropy constant = 6 × 10^5^ J/m^2^, spin polarization (*P*) = 0.7. The track thickness and width is *t*_nano_ = 0.4 nm and *w* = 60 nm, respectively. The results that are shown here are obtained with kerb thickness of *t*_kerb_ = 0.4 nm, and the results remain the same even when thicker kerbs are considered. Similar results are also obtained when a spacer layer is included underneath the kerbs to separate the kerb and the skyrmion layer.

## Additional Information

**How to cite this article**: Purnama, I. *et al*. Guided current-induced skyrmion motion in 1D potential well. *Sci. Rep.* **5**, 10620; doi: 10.1038/srep10620 (2015).

## Supplementary Material

Supplementary Figure 1

Supplementary Figure 2

Supplementary Figure 3

Supplementary Information

## References

[b1] Skyrme T. H. R. A unified field theory of mesons and baryons. Nucl. Phys. 31, 556–569 (1962).

[b2] Muhlbauer S. *et al.* Skyrmion lattice in a chiral magnet. Science 323, 915–919 (2009). 19213914 10.1126/science.1166767

[b3] Yu X. Z. *et al.* Real-space observation of a two-dimensional skyrmion crystal. Nature 465, 901–904 (2010). 20559382 10.1038/nature09124

[b4] Huang S. X. & Chien C. L. Extended skyrmion phase in FeGe (111) thin films. Phys. Rev. Lett. 108, 267201 (2012). 23005010 10.1103/PhysRevLett.108.267201

[b5] Heinze S. *et al.* Spontaneous atomic-scale magnetic skyrmion lattice in two dimensions. Nat. Phys. 7, 713–718 (2011).

[b6] Fert A. & Levy P. M. Role of anisotropic exchange interactions in determining the properties of spin-glasses. Phys. Rev. Lett. 44, 1538–1541 (1980).

[b7] Fert A. Magnetic and transport properties of metallic multilayers, Metallic Multilayers 59-60, 439 (1990).

[b8] Nagaosa N. Tokura Y. Topological properties and dynamics of magnetic skyrmions. Nat. Nanotechnol 8, 889–911 (2013). 10.1038/nnano.2013.243 24302027

[b9] Sampaio J., Cros V., Rohart S., Thiaville A. & Fert A. Nucleation, stability and current-induced motion of isolated magnetic skyrmions in nanostructures. Nat. Nanotechnol 8, 839–844 (2013). 24162000 10.1038/nnano.2013.210

[b10] Fert A., Cros V. & Sampaio J. Skyrmions on the track. Nat. Nanotechnol 8, 152–156 (2013). 23459548 10.1038/nnano.2013.29

[b11] Yu X. Z. *et al.* Skyrmion flow near room temperature in an ultralow current density. Nat. Commun. 3, 988 (2012). 22871807 10.1038/ncomms1990

[b12] Iwasaki J., Mochizuki M. & Nagaosa N. Universal current-velocity relation of skyrmion motion in chiral magnets. Nat. Commun. 4, 1463 (2013). 23403564 10.1038/ncomms2442

[b13] Schultz T. *et al.* Emergent electrodynamics of skyrmion in a chiral magnet. Nat. Phys. 8, 301–304 (2012).

[b14] Iwasaki J., Mochizuki M., Nagaosa N. Current-induced skyrmion dynamics in constricted geometries. Nat. Nanotechnol 8, 742–747 (2013). 24013132 10.1038/nnano.2013.176

[b15] Brataas A., Kent A. D. & Ohno H. Current-induced torques in magnetic materials. Nat. Mater. 11, 372 (2012). 22522637 10.1038/nmat3311

[b16] Thiaville A., Nakatani Y., Miltat J. & Suzuki Y. Micromagnetic understanding of current-driven domain wall motion in patterned nanowires. Europhys Lett. 69, 990 (2005).

[b17] Zhou Y. & Ezawa M. A reversible conversion between a skyrmion and a domain-wall pair in a junction geometry. Nat. Commun. 5, 4652 (2014). 25115977 10.1038/ncomms5652

[b18] Metaxas P. J. *et al.* High domain wall velocities via spin transfer torque using vertical current injection. Sci. Rep. 3, 1829 (2013). 23670402 10.1038/srep01829 PMC3653216

[b19] Khvalkovskiy A. *et al.* High domain wall velocities due to spin currents perpendicular to the plane. Phys. Rev. Lett. 102, 067206 (2009). 19257631 10.1103/PhysRevLett.102.067206

[b20] Vansteenkiste A. *et al.* The design and verification of mumax3”, AIP Adv. 4, 107133 (2014).

[b21] Rohart S. & Thiaville A. Skyrmion confinement in ultrathin film nanostructures in the presence of Dzyaloshinskii-Moriya interaction. Phys. Rev. B 88, 184422 (2013).

[b22] Romming N. *et al.* Writing and deleting single magnetic skyrmions, Science 341, 6146 (2013). 10.1126/science.1240573 23929977

[b23] Najafi M. *et al.* Proposal for a standard problem for micromagnetic simulations including spin-transfer torque. J. Appl. Phys. 105, 113914 (2009).

[b24] Slonczewski J. C. Current-driven excitation of magnetic multilayers. J. Magn. Magn. Mater. 159, L1–L7 (1996).

[b25] Xiao J., Zangwill A. & Stiles M. D. Boltzmann test of slonczewski’s theory of spin-transfer torque. Phys. Rev. B 70, 172405 (2004).

[b26] Zhang S. & Li Z. Roles of nonequilibrium conduction electrons on the magnetization dynamics of ferromagnets, Phys. Rev. Lett. 93, 127204 (2004). 15447304 10.1103/PhysRevLett.93.127204

[b27] Donahue M. J. & Porter D. G. National Institute of Standards and Technology, Interagency Report NISTIR, 6376 (1999 OOMMF user’s guide, version 1.0.).

[b28] Barman A. *et al.* Ultrafast magnetization dynamics in high perpendicular anisotropy [Co/Pt]_n_ multilayers. J. Appl. Phys. 101, 09D102 (2007).

[b29] Metaxasm P. J. *et al.* Creep and Flow Regimes of Magnetic Domain-Wall Motion in Ultrathin Pt/Co/Pt Films with Perpendicular Anisotropy. Phys. Rev. Lett. 99, 217208 (2007) 18233251 10.1103/PhysRevLett.99.217208

