# Supplementary figures and images for: Guided current-induced skyrmion motion in 1D potential well
Source: Sci Rep. 2015 May 29;5:10620. doi: 10.1038/srep10620 (PMC4448553; doi:10.1038/srep10620)

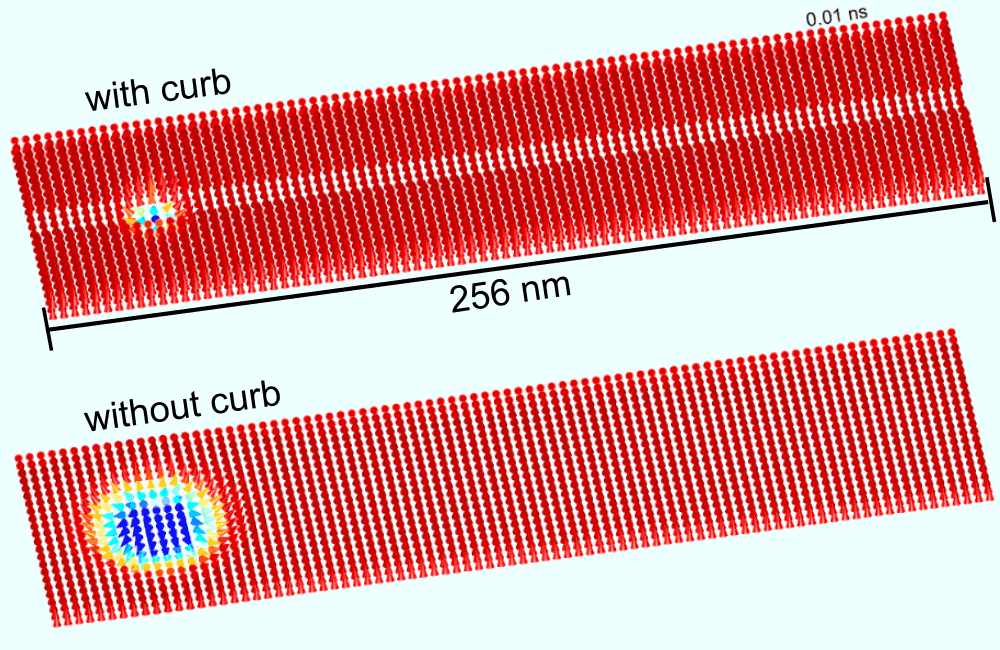

Supplement: Supplementary Figure 1 [file srep10620-s1.gif]

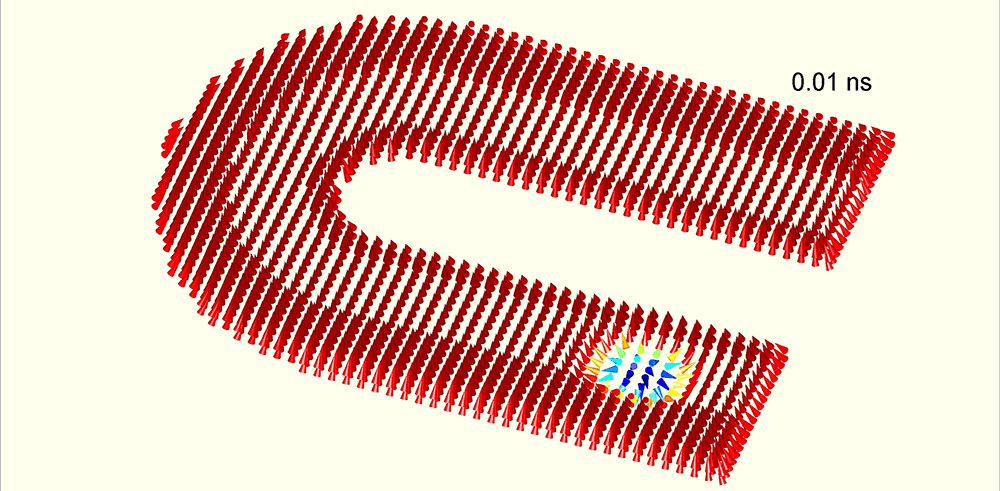

Supplement: Supplementary Figure 2 [file srep10620-s2.gif]

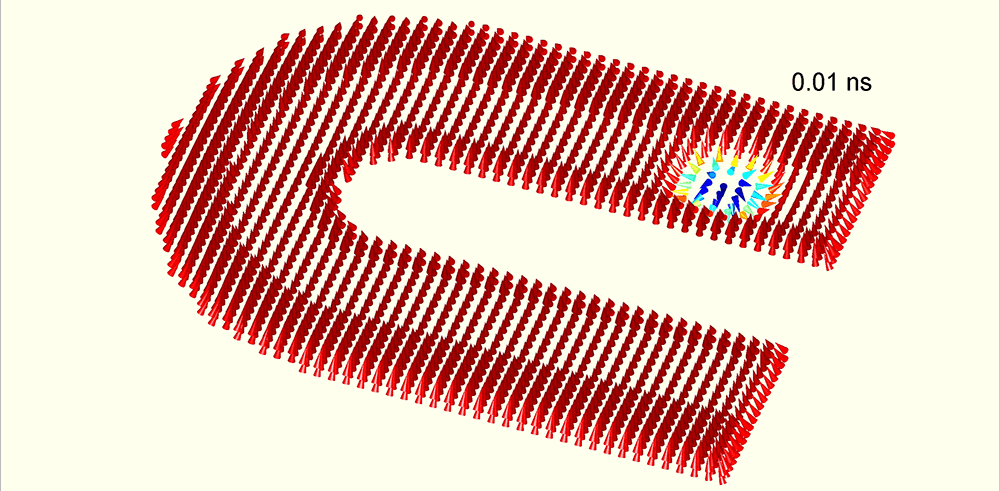

Supplement: Supplementary Figure 3 [file srep10620-s3.gif]
