# Supplementary material for: Guided current-induced skyrmion motion in 1D potential well
Source: Sci Rep. 2015 May 29;5:10620. doi: 10.1038/srep10620 (PMC4448553; doi:10.1038/srep10620)
Supplement: Supplementary Information [file srep10620-s4.pdf]

Supplementary Material Guide

Manuscript Title: Guided current-induced skyrmion motion in 1D potential well

Authors: I. Purnama, W. L. Gan, D. W. Wong, and W. S. Lew

Supplementary Material(s) that are included in this work:

Title: Supplementary Material

Description: Details of skyrmion driving under perpendicular current injection S1

Supplementary Figures S1 to S6

Title: Video 1

File name: skyrm compare.gif

Description: A video which shows the motion of a skyrmion on both unkerbed and kerbed track.

Title: Video 2

File name: L2R.gif

Description: A video which shows the motion of a skyrmion on a curved track with the current being applied along the clockwise direction.

Title: Video 3

File name: R2L.gif

Description: A video which shows the motion of a skyrmion on a curved track with the current being applied along the anticlockwise direction.

Supplementary Material

Guided current-induced skyrmion motion in 1D potential well

I. Purnama, W. L. Gan, D. W. Wong, and W.S. Lew*

School of Physical and Mathematical Sciences, Nanyang Technological University

21 Nanyang Link, Singapore 637371

*Corresponding author: wensiang@ntu.edu.sg

S1) **Skyrmion driving under perpendicular current injection**

In the perpendicular current setup, the skyrmion dynamics is mostly affected by the adiabatic contribution from the spin transfer torque [1, 2]. In the original manuscript, we have shown that the application of perpendicular current injection leads to the skyrmion getting enlarged as it moves within the ferromagnetic film.

We have performed additional simulations to provide further understanding of the skyrmion dynamics under perpendicular current injection. Fig. S1 shows the snapshots of the simulation when a skyrmion is driven on a film without kerbs. The result shows that the skyrmion is annihilated at the edges of the ferromagnet film when the edges are not kerbed, as suggested by the referee.

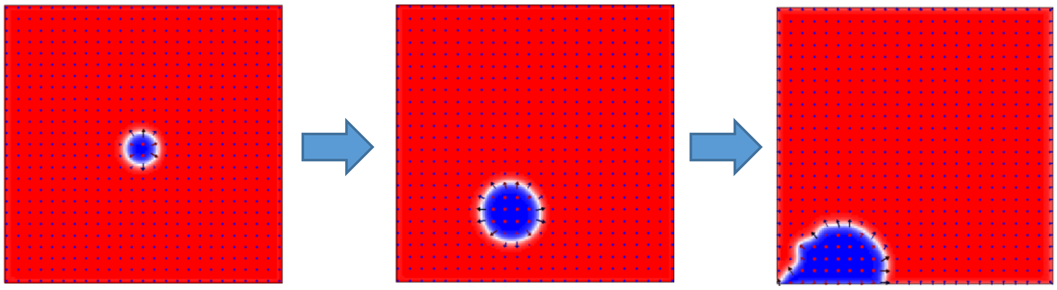

Fig. S1. *Snapshots of the simulations on a ferromagnetic film without kerbs.*

Fig. S2 shows snapshots of the simulations when the kerbs are incorporated to the ferromagnetic film. The result shows that the skyrmion annihilation is able to be prevented at the film edge due to the inclusion of the kerb.

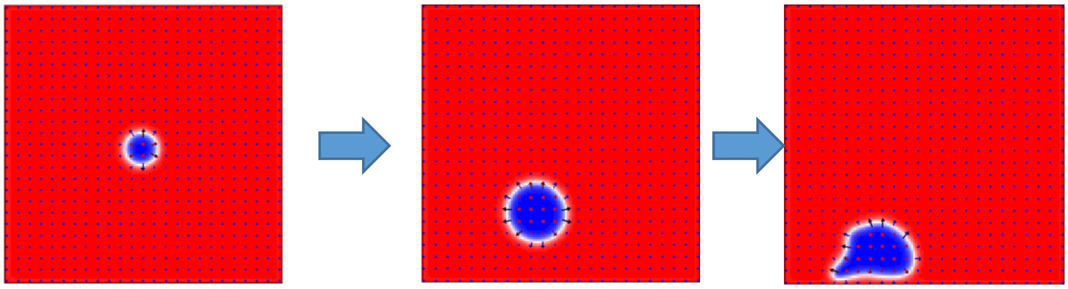

Fig. S2 *Snapshots of the simulations on a ferromagnetic layer with kerbed edges under perpendicular current injection. The magnetizations of the film at the lower edge are shown to remain pointing in the opposite direction as the skyrmion.*

When the distance between the kerbs is less than the skyrmion diameter, the skyrmion enlargement is suppressed by the kerbs, and the skyrmion adopts a more compact size, as shown by Fig. S3. The results also show that when the skyrmion hits the kerb, the speed component that is transverse to the kerb is negated, while the speed component that is parallel to the kerb remains. Consequently, this leads to the skyrmion moving parallel to the film edge under the application of perpendicular current injection, with the kerb acting as the guide.

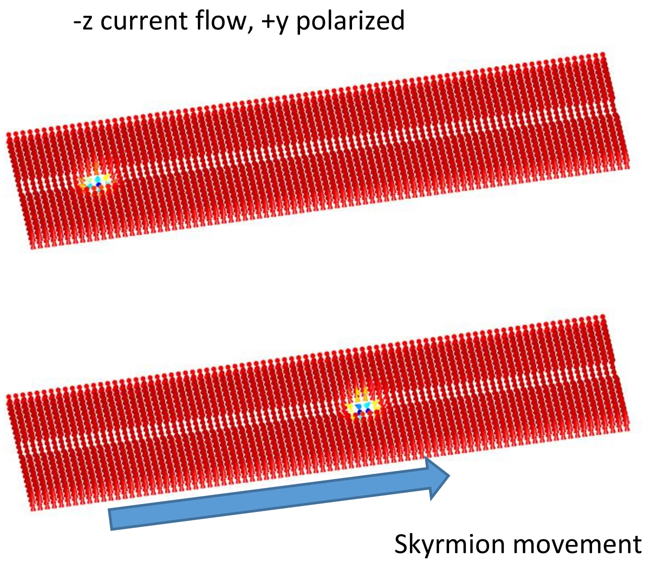

Fig. S3 *Snapshots of the simulations on a ferromagnetic track with kerbed edges under perpendicular current injection.*

Fig. S4 shows the skyrmion speed as functions of the kerb width (*s*) and the applied current density for both in-plane and perpendicular current setups. The result shows that in the perpendicular current setup, the skyrmion speed increases initially as the kerb width is increased. However, the skyrmions speed starts to drop when the kerb width is increased beyond 20 nm.

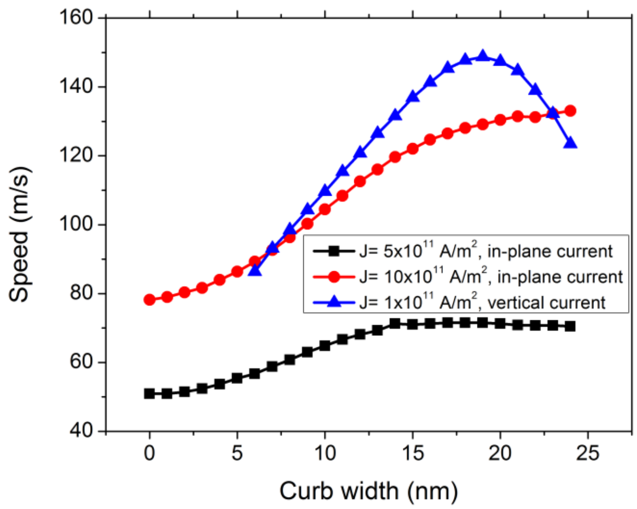

Fig. S4 *The skyrmion speed as a function of the kerb width for various applied current density.*

Fig. S5 shows the Spin-Transfer Torque (STT) that is acting on the skyrmion as a function of the kerb width. Here, we observe that for larger kerbs, the skyrmion is compressed and experiences an increased STT on the front and the back of the skyrmion, as indicated by the darker colours. The increase in the speed of the skyrmion can then be attributed to the increase in the STT. However, due to its smaller size, the total STT that is received by the skyrmion is reduced, as shown in Fig. S6. The total STT can be calculated by performing a line integral over the length of the skyrmion. The drop in the skyrmion speed under perpendicular current injection when the width of the kerbs is increased beyond 15 nm can then be attributed to the lower total STT that the skyrmion received.

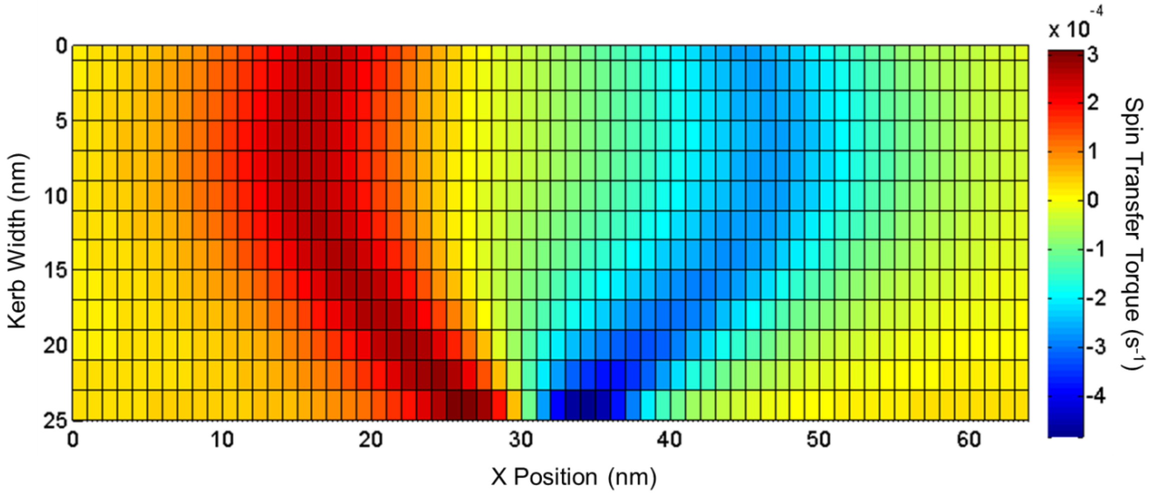

Fig. S5 *The perpendicular STT across the centre of the skyrmion for different kerb widths. The track has a width of 60 nm, and the STT is measured at the middle of the track.*

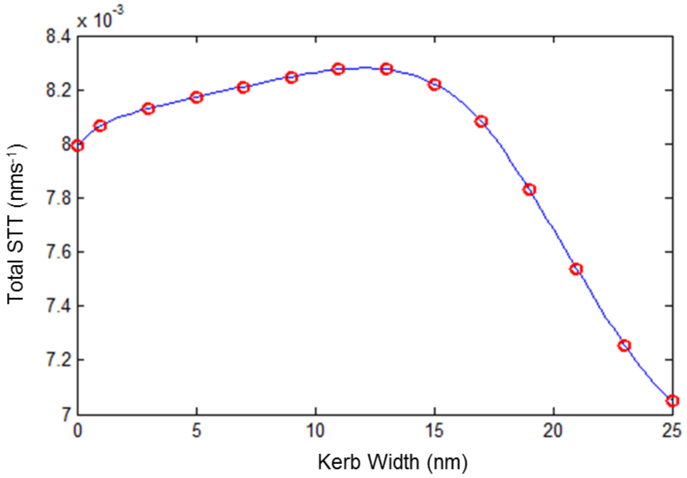

Fig. S6 *The total perpendicular STT across the centre of the skyrmion.*

References

1. Metaxas, P. J. *et al*. High domain wall velocities via spin transfer torque using vertical current injection. *Sci. Rep.* **3**, 1829 (2013).
2. Khvalkovskiy, A. *et al*. High domain wall velocities due to spin currents perpendicular to the plane. *Phys. Rev. Lett.* **102**, 067206 (2009).
